# Long-Term Safety and Usefulness of Mexiletine in a Large Cohort of Patients Affected by Non-dystrophic Myotonias

**DOI:** 10.3389/fneur.2020.00300

**Published:** 2020-05-20

**Authors:** Anna Modoni, Adele D'Amico, Guido Primiano, Fiorentino Capozzoli, Jean-François Desaphy, Mauro Lo Monaco

**Affiliations:** ^1^Department of Geriatric, Neurologic, Orthopedics and Head-Neck Science, Area of Neuroscience, Institute of Neurology, Fondazione Policlinico Universitario A. Gemelli, IRCCS, Rome, Italy; ^2^Unit of Neuromuscular and Neurodegenerative Diseases, Laboratory of Molecular Medicine, Department of Neurosciences, Bambino Gesú Children's Hospital, Rome, Italy; ^3^Department of Neurosciences, Italian National Institute of Health, Rome, Italy; ^4^Department of Biomedical Sciences and Human Oncology, University of Bari Aldo Moro, Polyclinic, Bari, Italy; ^5^MiA Onlus (“Miotonici in Associazione”), Portici, Italy

**Keywords:** mexiletine, non-dyspophyc myotonias, treatment tolerability, adverse effects, genotype-phenotype correlations

## Abstract

**Objective:** The aim of our study was to evaluate the long-term efficacy and safety of mexiletine in 112 patients affected by genetically confirmed non-dystrophic myotonias. The study was performed at the Neurophysiologic Division of Fondazione Policlinico Universitario A. Gemelli Istituto di Ricerca e Cura a Carattere Scientifico (IRCCS), Rome and the Children's Hospital Bambino Gesù, Rome.

**Methods:** The treatment was accepted by 59 patients according to clinical severity, individual needs, and concerns about a chronic medication. Forty-three patients were affected by recessive congenita myotonia, 11 by sodium channel myotonia, and five by dominant congenital myotonia. They underwent clinical examination before and after starting therapy, and Electromyography (EMG). A number of recessive myotonia patients underwent a protocol of repetitive nerve stimulations, for detecting and quantifying the transitory weakness, and a modified version of the Timed Up and Go test, to document and quantify the gait impairment.

**Results:** Treatment duration ranged from 1 month to 20 years and the daily dosages in adults ranged between 200 and 600 mg. No patient developed cardiac arrhythmias causing drug discontinuation. Mexiletine was suspended in 13 cases (22%); in three patients, affected by Sodium Channel myotonia, because flecainide showed better efficacy; in one patient because of a gastric cancer antecedent treatment; in four patients because of untreatable dyspepsia; and five patients considered the treatment not necessary.

**Conclusions:** In our experience, mexiletine is very useful and not expensive. We did not observe any hazarding cardiac arrhythmias. Dyspepsia was the most frequent dose-limiting side effect.

## Introduction

Non-dystrophic myotonias are due to loss-of-function mutations in the voltage-gated chloride ClC-1 channel, encoded by the *CLCN1* gene, or gain-of-function mutations in the voltage-gated sodium Nav1.4 channel, encoded by the *SCN4A* gene (1–3). These are rare disorders, with a prevalence of <1:100,000, characterized by clinical and electrophysiological myotonia, which is lifelong and impact quality of life. Today the drug of choice for treating myotonia is mexiletine, whatever the culprit gene (4–6). Mexiletine is a non-selective voltage-gated sodium channel blocker that belongs to the Class IB anti-arrhythmic drugs (6, 7).

In Italy, as in many other European countries, mexiletine was no longer available on the market, but since 2010 it can be obtained from the Military Chemical Pharmaceutical Plant of Florence (Stabilimento Chimico Farmaceutico Militare di Firenze) as a “named-patient” drug. Costs are entirely covered by the Italian National Health System.

Because of its activity on the heart, patients usually consider mexiletine a risky drug with potential cardiac side effects, a consideration that is often shared by primary care physicians despite literature data showing the absence of any significant change in Electrocardiogram (ECG) parameter or serious adverse cardiac event during long-term follow-up (6). On the other hand, some common non-cardiac side effects such as dyspepsia, nausea, heartburn, lightheadedness, and others are often dose-limiting (7).

The aims of this study were to evaluate the long-term efficacy and safety of mexiletine in 59 patients affected by non-dystrophic myotonias. The patients underwent clinical and neurophysiologic examination before and after treatment.

## Methods

Between 1999 and 2019, 112 patients affected by non-dystrophic myotonias have been followed at the Neurophysiologic Division of Fondazione Policlinico Universitario A. Gemelli IRCCS, Rome, and the Children's Hospital Bambino Gesù, Rome. Among these patients, 59 (33 males and 26 females) have accepted the suggestion of a symptomatic treatment and have been treated with mexiletine. Follow-up visits of treated patients have been scheduled every 6 months during the first year after starting treatment and then every year. Daily dosage and any side effects have been reported in each medical record.

### Clinical Examination

All the patients have undergone an in-depth clinical assessment. Clinical examination included searching for action myotonia wrist, eyelid, and tongue muscles, as well as percussion myotonia in the upper limbs (*extensor digitorum communis*) and lower limbs (*rectus femori*). In particular, the presence and duration of the myotonic phenomenon at wrist and eyelid was evaluated after a three-second forced closure. This maneuver was repeated five times subsequently in order to detect any paradoxical myotonia. Before looking for eyelid myotonia, the presence of lid-lag phenomenon was verified in patients lying in a supine position. The presence of transitory weakness was verified by asking the patient to exert a maximal voluntary contraction of *biceps brachii*: as soon as the muscle reached its peak force, a quick exhaustion developed, lasting until the muscle was allowed to relax and contract for four or five times. The muscle could fully recover only after such a warming up maneuver.

In addition, we examined the lower limbs motor difficulties that may occur due to either myotonia or transitory weakness by using a modified version of the Timed Up and Go test (8, 9). Specifically, patients were asked to run around a chair three times, first after rest (i.e., 5 min of sitting on the chair in a complete relaxed position, with extended legs) and then after warming up. We calculated the percentage difference between the time spent to perform the test at rest and after warming up as follows: (time at rest – time after warm-up) × 100/time at rest (chair test normal values: mean: 4.7%; SD: 8.0; *n* = 22; cut-off: 21%).

We also paid attention to muscle hypertrophy (grading 1–4), especially in neck and shoulders for NaM patients and lower limbs for MC patients.

### Neurophysiological Examination

All the patients were examined by needle EMG on *extensor digitorum communis* in order to detect myotonic discharges.

Clinical assessment oriented neurophysiologic evaluations, since different electromyographic patterns correlate with different pathogenic mechanism of muscle channelopathies (10–12). In particular, in our cohort of patients we performed the low-rate prolonged repetitive nerve stimulations (3Hz-RNS) to detect and quantify the transitory weakness of the intrinsic muscles of the hand (12). This test showed good tolerability and reproducibility, being performed before and after treatment. In short, the wrist ulnar nerve was stimulated at 3 Hz and the compound muscle action potential (CMAP) recorded from the *aductor digiti quinti* muscle. In some individuals, a transient depression of the CMAP amplitude developed during stimulation, reaching the nadir within about 30 s and recovering within 1 min of stimulation (12, 13). This transitory CMAP depression is considered the neurophysiological counterpart of the transitory weakness (14, 15).

### Genetic Analysis

Genetic analyses were performed based on clinical and electrophysiological findings. In presence of a prevalent neck or shoulder muscle hypertrophy together with eyelid myotonia, especially when paradoxical, strabismus, transitory diplopia, and/or referred adynamia, the mutational analysis was first on *SCN4A*. When the clinical features were mainly characterized by muscle hypertrophy of lower limbs, a more severe myotonia in the upper limbs compared to facial muscles, or in cases of transitory weakness, presence of mutations was first verified in *CLCN1*.

### Cardiac Evaluation

Before starting treatment with mexiletine, the patients performed a cardiac evaluation including a 12-derivations EKG and, if necessary, a 24 h-EKG monitoring. A cardiac follow-up was performed every year.

### Statistics

Average values are reported as mean ± SE. Statistical analysis was performed by using the paired Student's *t*-test.

Among patients affected by recessive congenital myotonia (RCM), we found several patients carrying the same mutation. Therefore, in each specific group of patients, we compared data obtained by 3Hz-RNS as well as Chair Test, also considering the different dosage of mexiletine in the three groups.

## Results

### Cohort Demographics

From the 112 patients affected by non-dystrophic myotonia with confirmed genetic diagnostic (55 males and 57 females, aged 2–78 years), two came from Albania, two from Romania, one from Egypt, one from Morocco, one from Guatemala, and all the others from Italy (26 from Southern, 68 from Central, and 11 from Northern Italy). The mutations in *CLCN1* gene encoding the ClC-1 chloride channel were the most frequent (77%), especially the recessive ones (54%).

All the patients were offered treatment and only 52% accepted.

### Mexiletine Dosage

Fifty-nine patients (33 males and 26 females) are or have been treated with mexiletine (Table 1). Until now, six of them are under 18 years and two of them are under 12. Patients under 12 are taking mexiletine at a dosage of 8 mg/Kg b.w. In adults and teenagers, the daily dosage range was between 200 and 600 mg according to clinical severity and individual needs.

**Table 1:** Demographics of the cohort of patients affected by non-dystrophic myotonias.

	**All the patients**	**RCM**	**DCM**	**NaM**
	112	60	26	26
Not treated	53 (47%)	17 (28%)	21 (81%)	15 (58%)
Treated with mexiletine	59 (53%)	43 (72%)	5 (19%)	11 (42%)
Situation on 2019-01-01				
Still on mexiletine	46 (78%)			
Drop-out	13 (22%)			
**Duration of treatment**				
<1 year				1 (7 drop-out)
1–4 years (mean range: 2.5 years)				14 (2 drop-out)
4–7 years (mean range: 5.5 years)				15 (1 drop-out)
>7 (until 20) years (mean range: 13.5 years)				16 (3 drop-out)

*RCM, Recessive Congenital Myotonia; DMC, Dominant Congenital Myotonia; NaM, Myotonia due to Sodium Channel (SCN4A) Mutation*.

Considering all the 59 treated patients, 43 (73%) were affected by recessive chloride channel myotonia (RCM), 11 (19%) had sodium channel myotonia (NaM), and five (8%) showed dominant chloride channel myotonia (DCM). Thus, 72% of all RCM patients, 19% of DCM, and 42% of NaM patients required treatment. Mexiletine treatment duration is reported in Table 1.

### Adverse Effects

Mexiletine has been discontinued in 13 cases (22%), in most cases within the first months of treatment (five patients during the first month and two within the sixth month of treatment).

Within the first year of treatment, 2 patients suspended treatment because of intolerable side effects, especially dyspepsia, while one patient decided to test another drug. Within 1 and 7 years of treatment, drug suspension was observed in three other patients due to side effects or personal motivations. Regarding the treatment period of 7–20 years, three patients affected by Na channel myotonia discontinued mexiletine to test flecainide. They experienced a dramatic clinical improvement with flecainide, as hypothesized by *in vitro* pharmacological studies (16–19). In three pediatric cases, a galenic formulation of mexiletine using sweetening drops was necessary because of bitter taste, when dosages lower than 200 mg were requested, or in presence of difficulty in swallowing capsules.

No cardiac arrhythmias have been detected. All the reported side effects of mexiletine are summarized in Table 2.

**Table 2:** Main adverse effects during mexiletine treatment.

**Number of treated patients (59)**	**Mexiletine side effects**	
29 (49%)	No side effects	
25	Dyspepsia	Mild Dyspepsia (no symptomatic drugs): 17
		Moderate Dyspepsia (dose limiting): 4
		Severe Dyspepsia (drop-out): 4
3	Insomnia	
1	Headache	
1	Dizziness	
1	Diarrhea	
1	Drowsiness	
3	“Intolerable” bitter taste	

Thus, four patients (7%) stopped mexiletine because of side effects, especially dyspepsia. Three patients (5%) shifted treatment to flecainide because of better efficacy. Five patients (8%) suspended the drug for personal motivations, while they were assuming mexiletine 200 mg/day (all these patients were affected by mild forms of myotonia and preferred not to establish a drug “addiction”), and one patient because of a gastric cancer pre-existent the treatment. In addition, four patients had to reduce daily drug doses from 600 to 400 mg/day because of the occurrence of dyspepsia.

### Genotype-Phenotype Correlations

In the RCM group, the most frequent *CLCN1* mutations were p.F167L (*n* = 15), the intronic c.180+3A>T (*n* = 9), and p.G190S (*n* = 6). Only eight patients (53%) carrying p.F167L took an anti-myotonic treatment, while all the patients carrying c.180+3A>T or p.G190S required treatment.

Before starting the drug, all these patients were examined using the 3Hz-RNS test. The Chair Test was performed in seven patients carrying p.F167L and in 12 patients carrying c.180+3A>T or p.G190S.

Table 3 shows the mean daily mexiletine dosage, the mean percent CMAP depression induced by 3Hz-RNS, and the mean percent time reduction measured by the Chair Test before and after warming up in all three groups of patients. We used a cut-off of −10% for 3Hz-RNS and +21% for the Chair Test (unpublished data). In the p.F167L group, only one patient resulted positive at both 3Hz-RNS and Chair Test, and only three out of 15 were positive to either 3Hz-RNS or Chair Test. In contrast, all the patients carrying p.G190S resulted positive to both tests. Likewise, all the patients carrying c.180+3A>T were positive to at least one of the two tests (8/9 positive to 3Hz-RNS and 4/6 to Chair test).

**Table 3:** Characterization of patients affected by recessive congenital myotonia carrying different *CLCN1* mutations.

**Genotype**	**F167L**	**G190S or 180+3A>T**	**F167L; G190S; 180+3A>T treated vs. untreated pts**
Number of patients	15	15	8 vs. 8
Mean mexiletine dosage (mg/die) (mean standard error)	160 (48)	460 (40), *p* < 0.001	
TD (mean standard error)	−4.5 (1.6)	−58 (5.9), *p* < 0.001	−18.4 (7.3) vs. −49.6 (12.5), *p* < 0.01
Chair test (mean standard error)	22.43 (4.3), *n* = 7	34.3 (3.2), *n* = 12, *p* < 0.05	22.8 (3.9) vs. 33.6 (6.2), *p* < 0.05

*In the last column comparison between treated and untreated patients (n = 8)*.

*TD, Nadir percent value of transitory CMAP depression during 3 Hz repetitive nerve stimulation. Chair test, three turns around a chair; percent amelioration after warm-up*.

*Statistical analysis was performed with paired Student's t-test*.

Importantly, we performed 3Hz-RNS and Chair Test in eight patients (two carrying p.F167L and six carrying p.G190S or c.180+3A>T) both before and during treatment: both tests showed a significant improvement during mexiletine treatment (Table 3).

## Discussion

It is worth noting that this study presents the limitations of a retrospective study and does not compare the treatment group to the non-treated patients. In addition, the population was not homogeneous due the different genes involved as well as their different mutations. Finally, the impact of myotonia on the limitation of the daily life activities is quite difficult to evaluate.

Notwithstanding, the study provides useful information on long-term mexiletine effects in a quite large cohort of myotonic patients followed up in a single center, taking also in account the rarity of these disorders. A multicenter study would allow examining a larger cohort but might also increase the risk of bias due to patients' evaluation in different centers by different physicians.

Mexiletine was a safe drug in most of the patients, as reported in other cohorts (4, 6). None of the treated patients developed cardiac arrhythmias or other severe side effects requiring drug discontinuation. Even in a pediatric patient aged three and affected by Wolf-Parkinson-White syndrome (WPW), mexiletine proved to be safe and the girl, now aged eight, is still on therapy. Although the treated population showed a predominance of patients with chloride channel mutations on those carrying sodium channel mutations, it is unlikely that safety was influenced by the genotype. Thus, we can assume a good profile of tolerance also in patients affected by NaM.

Considering the seven patients (12%) who discontinued mexiletine within the sixth month of treatment, five cases did not report any side effect and only one patient asked for another treatment. Thus, in our experience, the most important factor affecting the use of a symptomatic therapy is the patient's concern of taking medications “forever.” For instance, some patients adjusted the doses of mexiletine according to their physical activity. The five patients who decided to suspend any form of symptomatic treatment within the first month can be added to the 53 patients who refused to try any treatment from the very beginning, raising the number of “skeptical or not interested to any treatment” individuals to 58 (52%) and lowering the number of “motivated to treatment” patients to 54 (48%). All the “skepticals” were mildly affected, whereas severely affected myotonic patient never refused treatment.

Not tolerated side effects were responsible for drug discontinuation in four cases (7%) and dose reduction in other four patients (7%). In particular, dyspepsia was the most frequent dose-limiting side effect, as previously reported (4, 6, 7).

We observed that patients affected by RCM requested an anti-myotonic therapy more than patients affected by NaM. This observation seems to be in contrast with literature data regarding the greater intensity of myotonia in NaM (6). However, quantification of myotonia is very difficult and no data are available about correlations between daily doses of mexiletine and the entity of myotonic phenomenon.

It could be very difficult to merge non-dystrophic myotonias in a single group of disorders, considering the variability in severity and distribution of myotonia, which primarily affects the head-neck muscles in NaM and the limb muscles in RCM/DCM. Moreover, the possible association with other signs or symptoms such as paradoxical myotonia, transitory weakness, cold sensitivity, myalgia, or episodes of paralysis, and the warm-up phenomenon, contribute to make the clinical and neurophysiologic diagnosis, as well as the quantification of myotonia severity, very challenging. For instance, there is no single neurophysiologic test available to give an objective rating to the severity of the different clinical manifestations in different patients. Similarly, the rating scales, such as SF-36 (20) and INQoL (21), giving a global evaluation of self-reported health status and impact of disease on quality of life in myotonia (4, 22–25), do not always show comparable data, possibly because of different social and cultural background (22).

Considering all these limitations, we focused our attention on a selected group of patients affected by RCM carrying the most common *CLCN1* mutations (p.F167L, c.180+3A>T, p.G190S) with the aim of evaluating the sensitivity and specificity of our clinical and neurophysiologic tests, either in the assessment of clinical severity or in monitoring the efficacy of a treatment. Thus, we compared between these patient subgroups the results of 3Hz-RNS, which indirectly evaluates transitory weakness, and the Chair Test, which estimates the motor impairment due to myotonia alone or together with transitory weakness.

In experimental functional studies, the p.F167L mutation showed little effects on chloride channel function (26–28), while p.G190S causes a severe channel dysfunction “*in vitro*” (28–30). Likewise, we observed that both “*in vivo*” tests showed better results in patients carrying p.F167L compared with those carrying p.G190S or c.180+3A>T. Accordingly, only eight patients (53%) carrying p.F167L took an anti-myotonic treatment, while all the patients carrying c.180+3A>T or p.G190S required treatment (Table 3). In addition, the treated p.F167L patients showed much better results at both neurophysiological and clinical tests.

Last but not least, mexiletine is not only safe but, in Italy, is reasonably priced and easily available for adult treatment. Some difficulties can be experienced in the pediatric setting due to the unavailability of specific formulations for this age group, requiring the use of a galenic formulation of mexiletine sweetened drops. Thus, a formulation of mexiletine alternative to the capsule as syrup or drops would be very useful, especially for pediatric patients, considering the importance of an early treatment for a correct psychomotor development. Indeed, it has been recently highlighted that *SCN4A* variants may determine relevant symptoms in neonates, compromising respiratory and laryngeal function, and might be associated with Sudden Infant Death Syndrome (31, 32).

## Data Availability Statement

The datasets generated for this study are available on request to the corresponding author.

## Ethics Statement

The study was carried out in compliance with Helsinky Declaration, approved by the Ethic Committee-Fondazione Policlinico Universitario A. Gemelli, Rome, Italy (ethical approved ID 3075), and all patients gave a written informed consent authorizing storage and use of clinical data and DNA samples for any clinical research purpose about their data.

## Author Contributions

AM drafted the manuscript for intellectual content. AD'A analyzed data concerning pediatric patients. GP analyzed data with particular attention to adult patients. FC revised the text. JD critically revised the manuscript. ML conceptualized and designed the study.

## Conflict of Interest

The authors declare that the research was conducted in the absence of any commercial or financial relationships that could be construed as a potential conflict of interest.
